# Self-Reported vs. Measured Height, Weight, and BMI in Young Adults

**DOI:** 10.3390/ijerph15102216

**Published:** 2018-10-11

**Authors:** Melissa D. Olfert, Makenzie L. Barr, Camille M. Charlier, Oluremi A. Famodu, Wenjun Zhou, Anne E. Mathews, Carol Byrd-Bredbenner, Sarah E. Colby

**Affiliations:** 1 Davis College of Agriculture, Natural Resources and Design, Department of Animal & Nutritional Sciences, Agricultural Science Building, G025, West Virginia University, 333 Evansdale Dr., Morgantown, WV 26506, USA; mbarr6@mix.wvu.edu (M.L.B.); OluremiFamodu@tcomn.com (O.A.F.); 2 Clinical & Translational Science, School of Medicine, West Virginia University, 1 Medical Center Drive, Morgantown, WV 26506, USA; ccharlie@hsc.wvu.edu; 3 Business Analytics and Statistics, University of Tennessee, 916 Volunteer Blvd., Knoxville, TN 37996-0532, USA; wzhou4@utk.edu; 4 Food Science and Human Nutrition Department, Institute of Food and Agricultural Sciences, University of Florida, P.O. Box 110370, 359 FSHN Bldg., 572 Newell Dr., Gainesville, FL 32611-0370, USA; anne.mathews@ufl.edu; 5 Nutritional Sciences Department, Rutgers University, New Brunswick, NJ 08901, USA; bredbenner@sebs.rutgers.edu; 6 Department of Nutrition, University of Tennessee, 1215 W Cumberland Ave, 229 Jesse Harris Building, Knoxville, TN 37996, USA; scolby1@utk.edu

**Keywords:** self-report, anthropometrics, height, weight, young adults, BMI

## Abstract

Self-reported height and weight, if accurate, provide a simple and economical method to track changes in body weight over time. Literature suggests adults tend to under-report their own weight and that the gap between self-reported weight and actual weight increases with obesity. This study investigates the extent of discrepancy in self-reported height, weight, and subsequent Body Mass Index (BMI) versus actual measurements in young adults. Physically measured and self-reported height and weight were taken from 1562 students. Male students marginally overestimated height, while females were closer to target. Males, on average, closely self-reported weight. Self-reported anthropometrics remained statistically correlated to actual measures in both sexes. Categorical variables of calculated BMI from both self-reported and actual height and weight resulted in significant agreement for both sexes. Researcher measured BMI (via anthropometric height and weight) and sex were both found to have association with self-reported weight while only sex was related to height difference. Regression examining weight difference and BMI was significant, specifically with a negative slope indicating increased BMI led to increased underestimation of weight in both sexes. This study suggests self-reported anthropometric measurements in young adults can be used to calculate BMI for weight classification purposes. Further investigation is needed to better assess self-reported vs measured height and weight discrepancies across populations.

## 1. Introduction & Background

Obesity is a significant American public health concern and a recognized worldwide epidemic, with major medical and financial ramifications [1,2,3,4]. In the United States, the Centers for Disease Control (CDC) officially links obesity with serious chronic health conditions such as heart disease, type 2 diabetes, stroke, and several types of cancer including colon, pancreas, and breast cancers [5,6]. Furthermore, obesity is thought to cost approximately $147 billion annually in medical expenses alone [7]. The latest data available from the CDC estimates the obesity rate to be approximately 36.5% among American adults and 17% among American youth for the time period between 2011 and 2014 [8]. In light of the increasing prevalence and high cost of obesity, the need for intervention is high. However, to be feasible and effective on a large scale, intervention across the nation requires quick and reliable identification of both obese and overweight individuals. 

Body Mass Index (BMI) is a widely used measurement to examine weight status of the average population as it is an accessible and cost-effective tool, only requiring height and weight measurements in order to identify individuals as underweight, normal weight, overweight, obese, and severely obese [9]. Additional anthropometric measurements such as waist, hip, and neck circumferences are also used to assist with assessing overall weight and body fat distribution [10]. Various types of physical measurements (i.e., height, weight, BMI, waist circumference) are often self-reported for convenience, particularly in large sample studies, or in dissemination and implementation research where researchers are unable to visit participants to take measurements. Thus, self-reported body measurements tend to fall into one of two categories: self-measurements taken in the home environment and later reported to researchers or medical professionals, and self-reported without direct measurements, i.e., via surveys asking participants to provide a particular anthropometric measurement without access to a measuring tool, resulting in participants sharing an estimate of what they perceive that measurement to be at the time of the survey. 

Self-measurement reports have been found to be both valid and reliable when compared to measurements taken directly by researchers [11,12]. These self-reported anthropometric measurements have been shown to withstand the test of time and remain useful for health and epidemiological purposes for at least ten years [11]. Longitudinal work in healthcare is both sorely needed and notoriously difficult to fund [13]. As such, longevity of data validity is of great interest, especially when addressing chronic conditions such as obesity. Consequently, in dissemination and implementation research, utilization of measures that can be accurately taken without a researcher present is going to be vital. When disseminating a program for use across the world, the researchers who originated the program and data analyses would be unable to take time and funding to travel to each participant in the program and take reliable measurements such as anthropometrics. A solution to this would be through accurate self-reported measures.

However, discrepancies do still occur when those measurements are compared with those taken by trained professionals. One example of a source of inconsistency in measurements is the use of bathroom scales. Indeed, each participant typically uses their own private scale leading to data of a sample being collected using a wide variety of instruments. For example, digital home-scales have been found to provide more accurate and consistent measurements than dial scales [14]. Self-reported measurements via survey collection are much more commonly used than direct self-measurements, with both strengths and limitations. At this time, most surveys are still paper based but online versions are also valid options which will likely become more prominent in the future [15]. Regardless of survey medium, both men and women are overwhelmingly seen overestimating their height and underestimating their weight [12,16,17,18,19,20]. This underestimation of weight is more pronounced in women [17,21] and in overweight individuals regardless of gender [17,18,19,22]. However, it is noteworthy that recent studies appear to show that the inconsistencies in self-reported height and weight in obese individuals are actually diminishing, resulting in more accurate BMI data in what is arguably the population most at risk for weight-related health complications [23]. 

This study aimed to investigate self-reported versus measured height and weight, and subsequent BMI, in college freshmen students across the U.S. This data will expand on previous cohorts of young adults to examine a large cohort of current college students. 

## 2. Methods

This study analyzed a cross-section of data generated by the GetFruved project, a multi-institutional program funded by the United States Department of Agriculture (USDA) with the goal of identifying and improving lifestyle behaviors in college students. As part of this study, over 1500 college students across eight U.S. institutions provided their height and weight via an electronic survey. Anthropometric measurements were subsequently taken by trained personnel for comparison. All individuals were physically assessed at baseline (August 2015) to capture anthropometrics of height and weight. Height was measured via a Stadiometer (SECA 213) in a standing position with shoes removed, shoulders relaxed, facing forward with head and back facing the wall. Weight was measured with minimal clothing on, via a Tanita TBF-310GS Total Body Composition Analyzer. All measures were performed twice and averaged for reliability and accuracy. Self-reported measurements were provided in English units and thereafter converted to metric units by researchers. Metric units were also used for investigator-collected measurements. As eligibility criteria required participants to be 18 years or older, adult BMI measures were used [24]. BMI was calculated as weight (kg) divided by height squared (m^2^) by investigators for both self-reported and measured data. BMI was also categorized using international adult standards: underweight—BMI < 18.5, normal weight—BMI = 18.5 to 24.9, overweight—BMI = 25.0 to 29.9, and obese—BMI > 29.925. Differences between self-reported and measured height and weight were calculated and designated as height difference and weight difference.

Data collection also included students’ location (i.e., U.S. region and state), sex, race, and Appalachian status. The latter variable being selected due to the geographical distribution of participating institutions in the states of Alabama, Florida, Kansas, Maine, New York, Tennessee, South Dakota, and West Virginia. Note that demographic information was self-identified. 

Statistical analysis was completed using SAS and JMP software. Spearman’s Rho was selected to assess correlation between self-reported and actual measurements, i.e., between self-reported and measured height, self-reported and measured weight, and calculated self-reported and measured BMI. Additionally, BMI categories were tested for agreement using unweighted Kappa coefficient. Relationships between BMI, sex and differences between self-reported and measured height and weight were investigated using simple linear regression and ANCOVA.

## 3. Results

A total of 1562 subjects completed the survey. This sample consisted predominantly of females (68.12%), non-Appalachians (72.98%), and individuals identifying their race as “White” (56.40%). The sample was an average of 19.1 ± 1.2 years of age (range 18–28). The geographical locations of the participants were widespread, with Florida the only state representing more than 20% of the data at 25.5% of the sample. See Table 1 for additional breakdown of participants’ demographics.

Due to incomplete and missing data, all variables were not always available for each subject. Of the 1562 total participants, 1514 reported gender. Of the 1522 participants we have anthropometric measured height and weight, 442 males had measured BMI, and 419 males had self-reported BMI. Of females, 1034 had measured BMI and 864 had self-reported BMI. In overall participants, most young adults fell into the normal weight category (58.0%), followed by overweight (24.3%), obese (9.7%), and underweight (5.3%) for self-reported BMI. Similar distribution was observed for both sexes. Table 2. 

## 4. Accuracy of Self-Reported versus Measured Height, Weight, and BMI

A percentage of 30.4% (*n* = 413) of participants self-reported their height within the range of ±5.08 cm (2 inches) of their physically measured height. 75.1% (*n* = 996) of participants self-reported their weight within the range of ±2.3 kg (5 pounds) of their physically measured weight. Self-reported and measured BMI categories showed significant agreement for both sexes: males κ = 0.79 (95% CI, 0.73 to 0.84), *p* < 0.001, and females κ = 0.76 (95% CI, 0.72 to 0.80), *p* < 0.001. Note that this trend was seen across variables, with unweighted Kappa coefficient indicative of good to very good agreement for all but two categories (Native Hawaiian/Pacific Islander and Biracial, both components of the Race variable), as shown in Table 3. 

## 5. Effect of BMI on Self-Reported Height and Weight

**Figure 1:**
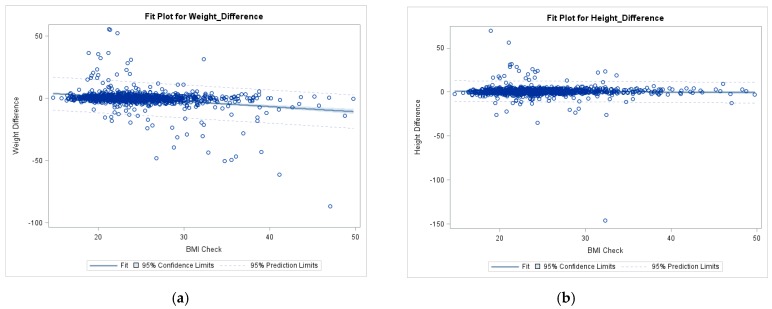
Simple linear regression of weight and height differences on BMI. (**a**) Plot of regression examining weight difference and BMI relationship. (**b**) Plot of regression examining height difference and BMI relationship.

**Figure 2:**
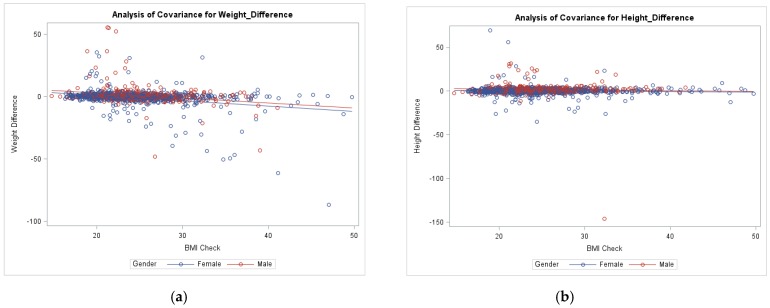
ANCOVA of weight and height differences on BMI for males and females. (**a**) ANCOVA model examining weight difference and BMI relationship among genders. (**b**) ANCOVA model examining height difference and BMI relationship among genders.

Preliminary screening for Linear and ANCOVA models found that all variables, except gender were found to be non-significant in the models and were ultimately removed to give the following Figure 1 and Figure 2. Participants measured BMI was found to have a relationship with the self-reported weight difference in the overall sample (*p* < 0.001), as shown in Figure 1 Simple Linear Regression model. Additionally, BMI and sex were both found to have a significant linear relationship with weight discrepancy (both *p* < 0.001) while only sex affected height difference (*p* < 0.001 versus *p* = 0.117 for BMI). The regression examining weight difference and BMI was significant, specifically with a negative slope (−0.41, *p* < 0.001) indicating that an increased BMI led to an increased underestimation of weight in both sexes. ANCOVA model included differences of self-reported height and weight to measured (i.e., Actual weight − self-reported weight = weight difference). ANCOVA model (Figure 2a) examined the main effect of gender on weight difference while controlling BMI (F (2, 1263) = 65.4, *p* < 0.0001). The second ANCOVA model (Figure 2b) examined the main effect of gender on height difference while controlling for BMI (F (2, 1439) = 36.9, *p* < 0.0001). Parallel female and male regression lines demonstrated this pattern, with females displaying stronger underestimation of weight than males, as shown in Figure 2. 

## 6. Discussion

Regression results detected that self-reported height and weight were strongly correlated to measured counterparts in this sample (all *p*-values < 0.001). This study’s findings are noteworthy in that self-reported height, weight and BMI were significantly correlated (r_s_ = 0.87–0.92) with measured height, weight and BMI across sex, race, Appalachian status, and geographical location (Table 3). Additionally, this large sample confirms previously documented slight overestimation of height by both sexes and underestimation of weight by females, with male participants displaying age-appropriate overestimation of their weight. However, these variances of estimation were minimal and similar to findings by Quick and Bowring. In a study of college students, Quick et al. (2015) found that self-reported height and weight were 93% accurate, lending additional support to the practice of using self-reported anthropometrics measurements in public health research [25]. In a study of young Australian adults, Bowring et al. (2012) concluded that, despite some evidence of over and under estimation, self-reported measurements were accurate enough to generate valid BMIs and subsequently correctly classify individuals as overweight or obese in the majority of cases [19]. Effect of BMI on self-reported weight was also consistent with literature as increased BMI correlated with increased underestimation of weight in both sexes. This does highlight the possible limitation of using self-reported anthropometrics in groups with high BMI. In a large sample such as the one in this study, any discrepancies caused by these subjects did not appear to affect the overall findings. However, further investigation focused on individuals with obesity and severe obesity only may yield different results. Nonetheless, this examination of young adult’s self-report of height and weight stands true among an additional cohort that utilizing self-reported measures in arenas such as a dissemination and implementation science can be valuable. In interventions where access to quality measuring tools or travel to participants may not be feasible, this method can substitute accurately. 

This study did have limitations, as its sample was solely made up of college students at large, American, state universities. Further investigation is needed to better assess self-reported versus measured height and weight discrepancies across populations. Various socio-economic statuses would be of particular value in light of the link between obesity and poverty, particularly in women [26] and adolescents [27]. Additionally, the extensive nature of this study resulted in measurements being taken at several institutions, with multiple researchers at each site. As such, the potential for inter-rater reliability cannot be discounted. The time of day at which measurements were taken may have also affected results as it may have differed from the time participants typically record their own weight. Finally, this project was based on voluntary surveys and anthropometrics assessments, and therefore contains an inherent volunteer bias. The sample was predominantly comprised of women and had limited racial diversity, which may not have been fully representative of the participating institutions.

These encouraging findings render assessing quality of self-reported data in various populations essential. However, when moving forward in research endeavors will involve data collection on participants across various parts of the world, including a thorough training for participants or leaders on providing accurate self-reported measures. This training could be through an online platform or sending a measuring tape for height and suggesting an affordable and reliable scale for everyone to have. 

## 7. Conclusions

This study suggests self-reported anthropometric measurements in young adults can be used to calculate BMI for weight classification purposes. This study also confirms the effect of BMI on underestimation of weight in both males and females. Further investigation is needed to better assess self-reported vs. measured height and weight discrepancies across various populations outside of young adults.

## Figures and Tables

**Table 1:** Demographic characteristics.

VARIABLE	Frequency (%)	Measured Ht (cm)	Self-Reported Ht (cm)	Ht *p*-Value	Measured Wt (kg)	Self-Reported Wt (kg)	Wt *p*-Value
	N = 1562	Mean ± SD	Mean ± SD		Mean ± SD	Mean ± SD	
**Sex**				<0.0001 *			<0.0001 *
Male	450 (29.8)	175.3 ± 7.7	177.3 ± 7.2		76.0 ± 15.2	76.8 ± 14.5	
Female	1064 (68.1)	164.7 ± 7.1	164.7 ± 7.5		65.7 ± 15.2	65.2 ± 14.4	
Did not answer	48 (3.1)	168.6 ± 10.2	169.1 ± 10.3		65.9 ± 12.3	65.3 ± 12.8	
**Race**				**0.1269**			**0.0635**
White	881 (56.4)	168.3 ± 8.6	169.0 ± 9.2		69.9 ± 15.9	69.9 ± 15.3	
Black or African American	161 (10.3)	167.0 ± 9.2	167.7 ± 9.8		69.6 ± 16.4	69.3 ± 14.5	
Asian	142 (9.1)	165.0 ± 8.9	165.6 ± 9.7		60.5 ± 12.4	60.9 ± 13.2	
American Indian/Alaska Native	16 (1.0)	170.7 ± 7.5	171.8 ± 8.4		77.6 ± 13.7	80.3 ± 14.1	
Native Hawaiian/other Pacific Islander	10 (0.6)	167.5 ± 9.3	168.7 ± 10.2		67.6 ± 14.1	69.5 ± 14.9	
Biracial	21 (1.3)	168.8 ± 10.3	165.8 ± 8.8		72.9 ± 18.3	68.5 ± 13.9	
Other	64 (4.1)	167.8 ± 9.6	168.8 ± 10.3		67.6 ± 15.7	66.3 ± 13.9	
Did not answer	267 (17.1)	168.2 ± 8.5	168.8 ± 9.2		67.9 ± 15.8	69.8 ± 17.1	
**State**				0.0248 *			<0.0001 *
Alabama	112 (7.2)	168.7 ± 8.7	163.4 ± 9.3		71.1 ± 18.8	70.4 ± 18.6	
Florida	399 (25.5)	166.1 ± 8.1	166.8 ± 8.5		64.1 ± 13.1	63.7 ± 12.4	
Kansas	111 (7.1)	169.1 ± 9.2	169.7 ± 9.9		72.9 ± 17.6	72.4 ± 17.6	
Maine	197 (12.6)	169.7 ± 8.5	169.2 ± 9.3		72.6 ± 17.5	71.4 ± 15.4	
New York	187 (12.0)	167.4 ± 9.7	169.0 ± 10.3		66.0 ± 13.1	67.4 ± 13.5	
South Dakota	136 (8.7)	167.8 ± 8.0	168.1 ± 8.5		69.7 ± 14.7	72.8 ± 16.3	
Tennessee	227 (14.5)	169.3 ± 9.3	170.4 ± 10.2		69.8 ± 15.3	72.5 ± 15.0	
West Virginia	193 (12.4)	167.1 ± 8.7	167.8 ± 9.4		70.6 ± 17.5	71.7 ± 16.8	
**Appalachian Status**				**0.1493**			**0.2510**
Appalachian	185 (11.8)	169.2 ± 9.2	169.8 ± 10.0		72.2 ± 17.6	71.7 ± 15.7	
Non-Appalachian	1140 (73.0)	167.8 ± 8.8	168.5 ± 9.4		68.7 ± 15.8	68.6 ± 15.2	
Did not answer	237 (15.2)	166.9 ± 8.4	167.6 ± 9.1		65.9 ± 14.1	67.0 ± 15.8	

Wilcoxon Rank Sum test use to examine relationship between categorical variables and the difference of self-reported and measured height and weight (i.e., measured height − self-reported height = height difference). * *p*-value less than 0.05 indicate significance.

**Table 2:** Body Mass Index (BMI) categories by sex.

Measure	Overall Sample	Males	Females	*p*-Value
	Mean ± SD	Mean ± SD	Mean ± SD	
**Measured Height**	167.85 ± 8.8	175.3 ± 7.7	164.7 ± 7.1	
**Self-Reported Height**	168.6 ± 9.6	177.3 ± 7.2	164.8 ± 7.9	
**Height Difference**	0.71 ± 3.9	2.0 ± 4.3	0.12 ± 3.5	<0.0001 *
**Measured Weight**	68.68 ± 15.8	76.0 ± 15.2	65.7 ± 15.2	
**Self-Reported Weight**	68.8 ± 15.4	76.8 ± 14.5	65.2 ± 14.4	
**Weight Difference**	−0.05 ± 7.0	1.1 ± 7.2	−0.61 ± 6.9	<0.0001 *
	**Frequency (%)**	**Frequency (%)**	**Frequency (%)**	
**Measured BMI Categories**	**(N = 1522)**	**(N = 442)**	**(N = 1034)**	
0—Underweight	83 (5.53)	15 (3.3)	63 (5.9)	
1—Normal Weight	906 (59.5)	256 (56.9)	624 (58.6)	
2—Overweight	380230 (25.0)	126 (28.0)	241 (22.7)	
3—Obese	152 153 (10.1)	45 (10.0)	106 (10.0)	
**Self-Report BMI Categories**	**(N = 1322)**	**(N = 419)**	**(N = 864)**	
0—Underweight	64 (4.8)	15 (3.6)	45 (5.2)	
1—Normal Weight	805 (60.9)	241 (57.5)	539 (62.4)	
2—Overweight	330 (25.0)	125 (29.8)	195 (22.6)	
3—Obese	123 (9.3)	38 (9.1)	85 (9.8)	

* Wilcoxon analyses for nonparametric height and weight data to test if means by gender differ; significant *p*-value < 0.05.

**Table 3:** Relationship of self-reported to measured BMI categories.

	Measured to Self-Reported BMI Categories	Agreement
**Overall Sample**	**κ = 0.77 (95% CI, 0.74 to 0.80), *p* < 0.0001**	**Good**
**Sex**		
Male	**κ = 0.79 (95% CI, 0.73 to 0.84), *p* < 0.0001**	**Good**
Female	**κ = 0.76 (95% CI, 0.72 to 0.80), *p* < 0.0001**	**Good**
**Race**		
American Indian/Alaskan Native	**κ = 0.66 (95% CI, 0.32 to 1.00), *p* < 0.0005**	**Adequate**
Asian	**κ = 0.72 (95% CI, 0.60 to 0.84), *p* < 0.0001**	**Good**
Black or African American	κ = 0.84 (95% CI, 0.75 to 0.92), *p* < 0.0001 *	**Very Good**
Native Hawaiian/Pacific Islander	κ = 0.44 (95% CI, -0.13 to 1.00), *p* < 0.0160	Poor
White	κ = 0.80 (95% CI, 0.77 to 0.84), *p* < 0.0001 *	**Very Good**
Biracial	κ = 0.42 (95% CI, 0.06 to 0.83), *p* < 0.0001	Poor
**Appalachian Status**		
Appalachian	**κ = 0.71 (95% CI, 0.61 to 0.80), *p* < 0.0001**	**Good**
Non-Appalachian	**κ = 0.79 (95% CI, 0.76 to 0.83), *p* < 0.0001**	**Good**
**State**		
AL	**κ = 0.69 (95% CI, 0.57 to 0.82), *p* < 0.0001**	**Adequate**
FL	**κ = 0.74 (95% CI, 0.68 to 0.81), *p* < 0.0001**	**Good**
KS	κ = 0.83 (95% CI, 0.74 to 0.93), *p* < 0.0001 *	**Very Good**
ME	**κ = 0.74 (95% CI, 0.65 to 0.83), *p* < 0.0001**	**Good**
NY	**κ = 0.77 (95% CI, 0.68 to 0.86), *p* < 0.0001**	**Good**
SD	κ = 0.82 (95% CI, 0.69 to 0.94), *p* < 0.0001 *	**Very Good**
TN	κ = 0.81 (95% CI, 0.73 to 0.89), *p* < 0.0001 *	**Very Good**
WV	**κ = 0.77 (95% CI, 0.67 to 0.87), *p* < 0.0001**	**Good**
**Region**		
Northeastern	**κ = 0.72 (95% CI, 0.65 to 0.78), *p* < 0.0001**	**Good**
Southeastern	κ = 0.80 (95% CI, 0.76 to 0.85), *p* < 0.0001 *	**Very Good**
Northwestern	**κ = 0.64 (95% CI, 0.24 to 1.00), *p* < 0.0028**	**Adequate**
Southwestern	κ = 0.95 (95% CI, 0.86 to 1.00), *p* < 0.0001 *	**Very Good**
Midwest	κ = 0.81 (95% CI, 0.74 to 0.88), *p* < 0.0001 *	**Very Good**

* Unweighted Kappa coefficient greater than 0.80, indicative of very good agreement; good agreement of 0.70 to 0.79, Adequate agreement 0.60 to 0.79.
